# Extending the Time Domain of Neuronal Silencing with Cryptophyte Anion Channelrhodopsins

**DOI:** 10.1523/ENEURO.0174-18.2018

**Published:** 2018-07-10

**Authors:** Elena G. Govorunova, Oleg A. Sineshchekov, Raheleh Hemmati, Roger Janz, Olivier Morelle, Michael Melkonian, Gane K.-S. Wong, John L. Spudich

**Affiliations:** 1 Center for Membrane Biology, Department of Biochemistry and Molecular Biology, The University of Texas Health Science Center at Houston, McGovern Medical School, Houston, TX 77030; 2 Department of Neurobiology and Anatomy, The University of Texas Health Science Center at Houston, McGovern Medical School, Houston, TX 77030; 3 Institute of Botany, Cologne Biocenter, University of Cologne, Cologne D-50674, Germany; 4 Departments of Biological Sciences and of Medicine, University of Alberta, Edmonton, AB T6G 2E1, Canada; 5 Beijing Genomics Institute-Shenzhen, Shenzhen 518083, China

**Keywords:** channelrhodopsins, chloride ion channels, neuronal inhibition, optogenetics

## Abstract

Optogenetic inhibition of specific neuronal types in the brain enables analysis of neural circuitry and is promising for the treatment of a number of neurological disorders. Anion channelrhodopsins (ACRs) from the cryptophyte alga *Guillardia theta* generate larger photocurrents than other available inhibitory optogenetic tools, but more rapid channels are needed for temporally precise inhibition, such as single-spike suppression, of high-frequency firing neurons. Faster ACRs have been reported, but their potential advantages for time-resolved inhibitory optogenetics have not so far been verified in neurons. We report RapACR, nicknamed so for “rapid,” an ACR from *Rhodomonas salina*, that exhibits channel half-closing times below 10 ms and achieves equivalent inhibition at 50-fold lower light intensity in lentivirally transduced cultured mouse hippocampal neurons as the second-generation engineered Cl^–^-conducting channelrhodopsin iC++. The upper limit of the time resolution of neuronal silencing with RapACR determined by measuring the dependence of spiking recovery after photoinhibition on the light intensity was calculated to be 100 Hz, whereas that with the faster of the two *G. theta* ACRs was 13 Hz. Further acceleration of RapACR channel kinetics was achieved by site-directed mutagenesis of a single residue in transmembrane helix 3 (Thr111 to Cys). We also show that mutation of another ACR (Cys to Ala at the same position) with a greatly extended lifetime of the channel open state acts as a bistable photochromic tool in mammalian neurons. These molecules extend the time domain of optogenetic neuronal silencing while retaining the high light sensitivity of *Guillardia* ACRs.

## Visual Abstract

**Figure:**
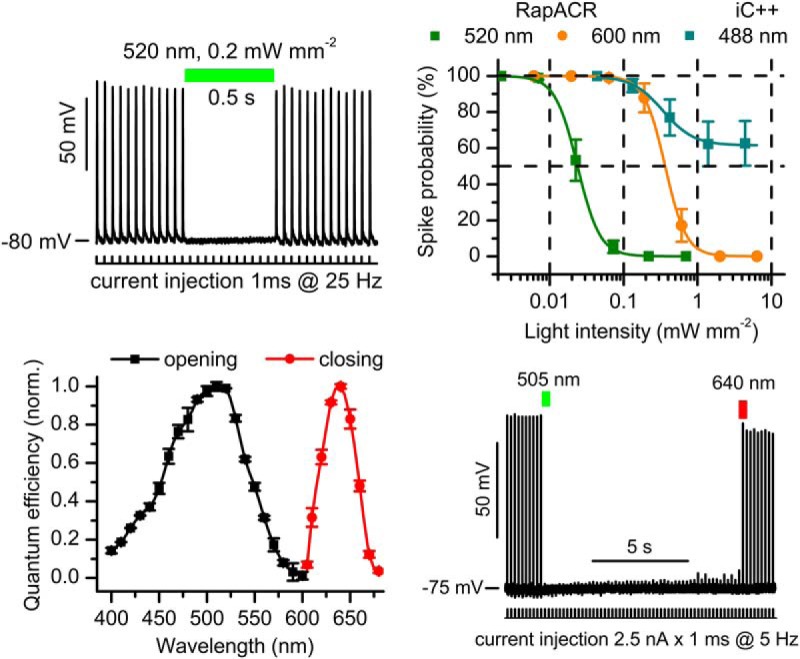

## Significance Statement

We report both fast and slow variants of cryptophyte anion channelrhodopsins (ACRs) that enable efficient neuronal silencing with light in the expanded time window of 10 ms to tens of seconds. These tools can be used at low expression levels to avoid possible detrimental effects on the physiology of recipient cells and help to minimize light intensities to prevent overheating of the tissue in optogenetic experiments. Their potential applications include analysis of neuronal circuitry, the slow closing channel variant enabling measurements requiring an open channel in the dark amenable to optical imaging, and the development of optogenetic treatments for neurologic diseases involving neuron hyperexcitability.

## Introduction

Control of neuronal activity with genetically targeted molecules (optogenetics) requires both actuators and inhibitors, to de- and hyperpolarize the membrane, respectively (Bernstein et al., 2012; Deisseroth, 2015; Emiliani et al., 2015). Until recently the only optogenetic tools available for inhibition of neuronal spiking had been rhodopsin ion pumps such as halorhodopsin and archaeorhodopsin that transport Cl^–^ in or H^+^ out of the cell, respectively (Zhang et al., 2007; Chow et al., 2010; Chuong et al., 2014). However, the pumps translocate at most only one charge per captured photon and therefore their optogenetic use typically requires high light intensities (10–20 mW mm^−2^; Maurice et al., 2015; Parker et al., 2016; Arguello et al., 2017).

Ion channels form a continuous hydrophilic pore in the lipid bilayer and are therefore much more efficient for manipulation of the membrane potential than ion pumps. Mature neurons maintain a steep inwardly directed Cl^–^ gradient across their somatic membrane (Price and Trussell, 2006; Khirug et al., 2008), so opening of Cl^–^ channels, such as endogenous ionotropic GABA receptors, hyperpolarize the membrane. Therefore, considerable efforts have been invested in converting cation channelrhodopsins (CCRs) from chlorophyte algae into Cl^–^ channels, yielding two principal variants (Berndt et al., 2014; Wietek et al., 2014). Continued engineering efforts resulted in second-generation tools by eliminating their residual permeability for H^+^ (Wietek et al., 2015; Berndt et al., 2016) and modification of their kinetics and spectral sensitivity (Wietek et al., 2017).

Meanwhile, two natural anion channelrhodopsins (ACRs) with unitary conductance that exceeds that of any previously known channelrhodopsins were found in the cryptophyte alga *Guillardia theta* (Govorunova et al., 2015). These proteins have been shown to require less light than rhodopsin pumps for inhibition of spiking in cultured rodent neurons (Govorunova et al., 2015), and behavioral responses in *Caenorhabditis elegans* (Bergs et al., 2018) and *Drosophila* (Mohammad et al., 2017). Furthermore, *Gt*ACRs did not produce rebound effects (Wei et al., 2018), observed when using rhodopsin pumps (Chuong et al., 2014; Mahn et al., 2016). *Gt*ACR2 enabled neuronal silencing at 20-fold lower light intensity with 65-fold better time resolution than that required by the most light-sensitive Cl^–^-conducting CCR mutants available at the time (Govorunova et al., 2015), but side-by-side comparison of natural ACRs with the second-generation engineered silencing tools has yet to be conducted.

Closing of *Gt*ACR channels requires tens to hundreds of milliseconds, which limits their temporal precision for silencing of neuronal types that fire at high frequencies. In a screen of natural ACR homologs, a faster molecule, named ZipACR, has been found, but its light sensitivity as a neuronal silencer has not been determined (Govorunova et al., 2017b).

In long-term optogenetic experiments, continuous illumination may lead to considerable heating of the tissue (Wiegert et al., 2017). One possible solution of this problem is to use “step-function tools,” i.e., light-gated channels that remain open for seconds after the light is switched off. Cl^–^-conducting CCR mutants have been converted into such tools (Berndt et al., 2014; Wietek et al., 2014) by mutagenetic disruption of the residues that form the “DC gate” (an interhelical hydrogen bond between Cys128 and Asp156, *Cr*ChR2 numbering; Nack et al., 2010). Mutation of the Cys128 homolog in *Gt*ACR1 to Ala led to a dramatic decrease in the rates of the second phase of channel closing and decay of the M intermediate of the photocycle (Sineshchekov et al., 2015, 2016). Expression of the *Gt*ACR1_C102A mutant in the body wall muscles of *C. elegans* enabled their relaxation on illumination (Bergs et al., 2018), but this mutant had not so far been tested in mammalian neurons.

In this study, we report a new ACR from the cryptophyte alga *Rhodomonas salina*, nicknamed RapACR, that provides 3–8-fold higher time resolution of neuronal inhibition (depending on the light intensity and membrane voltage) than the previously used *Gt*ACR2, while exhibiting a similar light sensitivity. A side-by-side test revealed that RapACR caused the same photoinduced rheobase shift at ∼50 times lower light intensity than the second-generation light-gated CCR-derived Cl^–^ channel iC++. We found that the C102A mutation increased the light sensitivity of *Gt*ACR1 ∼7-fold and permitted rapid restoration of neuronal firing by a pulse of red light. These findings provide strategies for using natural ACRs as inhibitory optogenetic tools and demonstrate their versatility.

## Materials and Methods

### Identification of ACR homologs in algal transcriptomes

ACR homologs were identified by searching over 2000 transcriptomes obtained from two sequencing projects, the MMETS project (Keeling et al., 2014) and 1KP project (Matasci et al., 2014) using probabilistic inference methods based on profile hidden Markov models (profile HMMs) implemented in HMMER software (version 3.1b2; Eddy, 2011). HMM profiles were built from previously known ACR sequences using default parameters and refined on functional testing of ACR homologs by electrophysiological measurements. Search procedures were automated with Python 2.7 and the Biopython module (Cock et al., 2009).

### Molecular cloning

DNA polynucleotides encoding the transmembrane domains of transcripts showing homology to previously known ACRs optimized for human codon usage were synthesized (GenScript) and cloned into the mammalian expression vector pcDNA3.1 (Life Technologies) in frame with an enhanced yellow fluorescent protein (EYFP) tag for expression in human embryonic kidney (HEK293) cells. The sequence information was deposited in GenBank. For expression in neurons, ACR fusion constructs were transferred to the pFUGW vector backbone (Lois et al., 2002). Mutants were generated using QuikChange XL kit (Agilent Technologies) and verified by sequencing. The polynucleotide sequence encoding iC++ was synthesized (GenScript) according to Berndt et al. (2016), fused to EYFP and cloned in the pcDNA3.1 and pFUGW vectors, as described for cryptophyte ACRs.

### HEK293 transfection and recording

HEK293 cells were transfected using the ScreenFectA transfection reagent (Waco Chemicals USA). All-*trans*-retinal (Sigma) was added at the final concentration of 4 µM immediately after transfection. Transfection with each tested ACR variant was repeated at least in three different batches of culture, and the results obtained in cells from all batches were pooled together.

Photocurrents were recorded 48–72 h after transfection in the whole-cell voltage clamp mode with an Axopatch 200B amplifier (Molecular Devices) using the 2-kHz low-pass Bessel filter. The signals were digitized at 5 kHz with a Digidata 1440A using pClamp 10 software (both from Molecular Devices). Patch pipettes with resistances of 2–5 MΩ were fabricated from borosilicate glass. Solution compositions are listed in Table 1. A 4 M KCl bridge was used in all experiments, and possible diffusion of Cl^–^ from the bridge to the bath was minimized by frequent replacement of the bath solution with fresh buffer. Individual transfected HEK293 cells were selected for patching by inspecting their tag fluorescence; non-fluorescent cells were excluded. Series resistance was periodically checked during recording, and experiments showing >20% increase were discarded. Data were also excluded if a gigaohm seal was lost during recording. Cells in which we could not establish a gigaohm seal were automatically excluded from measurements.

**Table 1.** Compositions of pipette and bath solutions and liquid junction potentials in experiments with HEK293 cells

	NaCl	KCl	CaCl_2_	MgCl_2_	Na_2_EGTA	HEPES	Glucose	NaAsp	pH	LJP
Pipette standard	—	126	0.5	2	5	25	—	—	7.4	—
Bath standard	150	—	1.8	1	—	10	5	—	7.4	4.7
Bath Asp	—	—	1.8	1	—	10	5	150	7.4	-7

Asp, aspartate; EGTA, ethylene glycol tetraacetic acid; HEPES, 4-(2-hydroxyethyl)-1-piperazineethanesulfonic acid; LJP, liquid junction potential. All concentrations are in mM.

Continuous light pulses were provided by a Polychrome V light source (T.I.L.L. Photonics GmbH) at the half-bandwidth 15 nm in combination with a mechanical shutter (Uniblitz Model LS6, Vincent Associates; half-opening time 0.5 ms). The light intensity was attenuated with neutral density filters. The maximal available quantum density at the focal plane of the 40× objective was measured with a piezo detector and ranged from 4.6 (400 nm) to 8.5 (470 nm) mW mm^−2^. For measurements of the dependence of photocurrents on the light intensity, the dark interval between subsequent light pulses was 30 s for all tested variants except the *Gt*ACR1_C102X mutants, for which it was 180 s because of their slow current decay. For measurements of the action spectra cells were illuminated with short (50 ms) pulses of monochromatic (half-bandwidth 10 nm) light of low intensity within the linear range of the dependence. To avoid possible adaptation during measurements, the spectral sensitivity in each cell was scanned with 10-nm intervals first from the shortest wavelength to the longest and then again in the reversed order.

To determine reversal potentials, we recorded in each cell a series of photocurrent traces in response to 1-s light pulses at the holding potentials changed in 20-mV increments from -60 to 60 mV at the amplifier output. The measurements were repeated first using standard solutions, and then on partial substitution of Asp^-^ for Cl^–^ in the bath (Table 1). One value of the reversal potential was obtained per one cell. All measurements were conducted at room temperature (25°C).

### Lentivirus production, titration, and delivery

Lentivirus was produced in HEK293FT cells (Invitrogen) by triple-transfection with the plasmids pCMV-VSVG, pΔ8.9 and corresponding channelrhodopsin-EYFP fusions in the pFUGW backbone using Lipofectamine 2000 (ThermoFisher). The virus was purified from the medium by ultracentrifugation at 17,000 rpm in an SW32 rotor for 2 h. Viral titers where determined by counting the percentage of fluorescent HEK293 cells in monolayers transduced with serially diluted virus preparations. The titers of concentrated virus were between 3 × 10^5^ and 5 × 10^6^ infectious units (IU)/ml. Neurons were transduced 1 d after plating with 10-100 µl of concentrated virus per slide to yield >90% of neurons to become fluorescent.

### Neuronal cultivation and recording

Mouse hippocampi (strain E18) were purchased from BrainBits, digested by papain to release individual neurons that were then cultured on top of a glia feeder layer in NbActiv4 medium (BrainBits) supplemented with all-*trans* retinal (0.4 µM). BrainBits collects the brains using procedures that are approved by their Institutional Animal Use and Care Committee. Patch clamp measurements were conducted 8–14 d after transduction. The same photoexcitation source and measuring setup was used as described above for HEK293 cells, except that the compositions of solutions were as listed in Table 2.

**Table 2.** Compositions of pipette and bath solutions and liquid junction potentials in experiments with neurons

	K gluconate	KCl	NaCl	CaCl_2_	MgCl_2_	HEPES	Glucose	pH	LJP
Pipette solution	135	—	—	—	2	—	—	7.2	16.5
Bath Tyrode’s solution	—	2	125	3	1	25	30	7.3	—

HEPES, 4-(2-hydroxyethyl)-1-piperazineethanesulfonic acid; LJP, liquid junction potential. All concentrations are in mM.

Individual neurons were randomly selected for patching without inspecting their tag fluorescence. Neurons in which we could not establish a gigaohm seal or those exhibiting >20% increase in access resistance during recording were excluded from measurements, as were those in which the seal broke during recording. Spiking was evoked by somatic current injection and recorded in current clamp mode. For measurements of the dependence of spiking probability on the light intensity, we injected 1-ms current pulses of 2.5 nA. Unless otherwise stated, the frequency of electrical stimulation was 5 Hz, because a subpopulation of rodent pyramidal hippocampal neurons generates high-frequency bursts of spikes on injection of a single short current pulse (Graves et al., 2012), which interferes with spiking probability measurements. At 5-Hz stimulation, the membrane voltage returned to the resting level before injection of each subsequent current pulse in all tested neurons. The duration of illumination was 2 s for each ACR variant, except the slow mutant *Gt*ACR1_C102A, for which it was 17 s. To avoid possible adaptation during measurements of light sensitivity, in each neuron, a series of light pulses was applied with 30-s dark intervals for all tested ACR variants, except *Gt*ACR1_C102A, for which the dark interval was 180 s, first from the lowest to the highest intensity and then again in the reversed order. For rheobase measurements neurons were injected with a current ramp (0–2 nA, 1 s) in the dark or 500 ms after the onset of actinic illumination. We found that the rheobase magnitude strongly depended on the duration of cultivation (days after transduction); therefore, neuronal cultures of the matched age were used for comparisons between different rhodopsin variants. For spiking recovery measurements, a single 1-ms current pulse was applied per each recorded trace at an incrementally varied time interval after the end of a 500-ms light pulse. Measurements in each neuron started with injecting current at the time 0, i.e., at the very end of the light pulse. If its spiking was inhibited at the given light intensity, the neuron was repetitively stimulated with an incrementally increased time interval between the light stimulus and current pulse using a 30-s dark adaptation period between subsequent traces.

### Immunofluorescence microscopy

Hippocampal neurons at day 14 post-transduction were fixed in 4% paraformaldehyde in PBS, washed, permeabilized (0.3% Triton X-100 in PBS), washed once again, and blocked with 2% goat serum in PBS for 30 min. The fixed permeabilized neurons were incubated with polyclonal rabbit anti-GFP antibody (1:500; catalog #132002, Synaptic Systems) and either guinea pig anti-MAP2 (microtubule associated protein 2) antibody (1:1000; catalog #188004, Synaptic Systems) or mouse anti-SV2 (synaptic vesicle glycoprotein 2) antibody (1:100; Registry ID: AB 2315387, Developmental Studies Hybridoma Bank) at 4°C overnight. For secondary staining, the preparations were incubated with Alexa Fluor 488-conjugated goat anti-rabbit antibody (catalog #A11034, Invitrogen) and Alexa Fluor 568-conjugated goat anti-guinea pig antibody (catalog #A11075, Invitrogen), or Alexa Fluor 647-conjugated goat anti-mouse antibody (catalog #A21236, Invitrogen) at room temperature for 1 h, after which the samples were washed and mounted with ProLongDiamond Antifade mounting medium (Invitrogen). Images were taken with ZEN software using an LSM 510 META laser scanning microscope (Carl Zeiss). The laser wavelengths were 488 nm (KrAr), 543 nm (HeNe), and 633 nm (HeNe). The emission filters LP 650, BP 490-510, and BP 560-615 were used.

### Data analysis

#### Estimation of mean photocurrent amplitudes, the degree of photocurrent inactivation, electrical charge transferred during illumination, and half-rise and half-decay times

From each cell, one current trace was recorded in the voltage clamp mode, and the baseline measured before illumination was subtracted using Clampfit software (a subroutine of pClamp). The same software was used to measure the peak current amplitude with a cursor, and to measure the stationary amplitude by averaging the data points during the last 100 ms of illumination. The degree of photocurrent inactivation was calculated by subtraction of the stationary value from the peak value, division by the peak value and multiplication by 100%. The amount of charge transferred across the membrane during illumination was calculated as the area (integral) under the current trace using Clampfit software. The rise and decay of ACR photocurrents are multiexponential (Sineshchekov et al., 2015); therefore, to characterize their kinetics, we calculated the half-rise and half-decay times, rather than their time constants, using Clampfit software. For each patched cell, there was one half-rise and one half-decay value. We averaged the photocurrent amplitude values, the degree of inactivation, the amount of charge and half-rise and half-decay times across all tested cells and calculated the SEM (the reported *n* values indicate the number of tested cells). The data points obtained in individual cells are shown in the respective figures as empty circles.

#### Determination of the photocurrent action spectra

In each cell, a response to illumination at each wavelength was measured at least twice in a symmetrical fashion, as described above. In each trace, the initial slope of photocurrent was assessed from the mean amplitude of the signal recorded during its close to the linear rise, usually during the first 5–15 ms depending on the current rate. The spectral data sets obtained in all scans (the number of which is indicated in the figure legend) were pooled together (because the differences between individual cells in ACR expression levels or patch parameters were not expected to influence ACR spectral properties), normalized to the maximal value and averaged to produce the mean and SEM values (the reported *n* values indicate the number of spectral scans). The mean and SEM values were then corrected for quantum density measured for each wavelength. The positions of the spectral maxima were determined using B-spline approximation of the data points in Origin 7 (OriginLab Corporation).

#### Calculation of the reversal potentials

In each current trace from a series measured at incremental holding voltages as described above, we calculated the peak value, as described above, transferred the obtained values to Origin 7 software and plotted them against the holding potential values corrected for liquid junction potentials calculated using the Clampex built-in LJP calculator (Barry, 1994). The reversal potential (E_rev_; the value at which the line connecting the data points crossed the *x*-axis) was determined using Origin software. The value obtained in the standard bath was then subtracted from that in the Asp^-^ bath thus yielding the value of the E_rev_ shift caused by Asp^-^ substitution. For each cell, one value of the E_rev_ shift was obtained. The shift values were averaged across cells to produce the mean and SEM values (the reported *n* values indicate the number of tested cells). The data points obtained in individual cells are shown as empty circles.

#### Determination of spiking probability

Spiking probability was determined by counting the number of fully developed spikes (with the amplitude >95% of that in the dark) during 2-s illumination periods using Clampfit software. For each neuron, the numbers of spikes during two illumination periods of the same intensity applied in a symmetrical fashion, as described above, were summarized, divided by the total number of applied electrical pulses (20) and multiplied by 100%. The intensity of half-inhibition for the slow *Gt*ACR1_C102A mutant was calculated using the last 2 s of the 17-s light pulse. For each light intensity, one percentage value was obtained for each neuron. The data were then averaged across all neurons to produce the mean and SEM values (the reported *n* values indicate the number of tested neurons). Logistic functions were then fit to the data sets measured at each wavelength using Origin software.

#### Rheobase measurements

Rheobase values (the amplitude of injected current at the start of the first evoked spike) were measured from the recorded traces with a cursor using Clampfit software. A single value was obtained for each tested light intensity in each neuron. Then the data were averaged across all neurons to produce the mean and SEM values (the reported *n* values indicate the number of tested neurons).

#### Spiking recovery analysis

For each neuron, a single series of traces was recorded for each tested light intensity, as described above. For comparison of the recovery times in neurons transfected with RapACR and *Gt*ACR2, only neurons for which 1% was the minimal light intensity that fully inhibited spiking were selected. The spike amplitude at each time point in each trace was measured using Clampfit software. The data were averaged across neurons to produce the mean and SEM values (the reported *n* values indicate the number of tested cells) and plotted against time using Origin software. After that, the value of the half-recovery time at each intensity was calculated for each neuron, averaged across neurons to produce the mean and SEM values (the reported *n* values indicate the number of tested cells) and plotted against the light intensity.

#### Image analysis

To quantify RapACR membrane targeting, lines were drawn through the center of cell images, and the fluorescence density profiles were calculated and analyzed using ImageJ 1.51j8 (National Institutes of Health). Primary dendrites from randomly selected neurons were identified as MAP2-positive branches originating from the soma and crossing the circumference of the 70-µm radius. Synapses (identified by SV2 staining) were quantified using Carl Zeiss ZEN microscope software in five randomly picked dendritic stretches (length, 20 µm) and averaging them for each neuron. From each neuron, one average dendrite number and one synapse number were derived, after which these numbers were averaged across all neurons.

#### Sample size determination

The sample size was estimated from previous experience, as recommended by the NIH guidelines (Dell et al., 2002).

#### Randomization

We presume that animals sacrificed for isolation of neurons were randomly selected by BrainBits. HEK293 and neuronal cultures were randomly assigned to experimental groups.

#### Statistics

Statistical analysis was performed using IBM SPSS Statistics software, and the selection of statistical tests was based on previously published studies. Normality and equal variances of the data were not assumed, and therefore non-parametric statistical tests were used; *p* > 0.05 was considered not significant. For pairwise comparisons of independent data sets the two-sided Mann–Whitney test was used, and for comparisons of multiple independent data sets, the Kruskal–Wallis test with Bonferroni correction (the similarity of distributions was assessed by visual inspection of a boxplot). When no specific statistical hypothesis was tested, descriptive statistics was reported as mean ± SEM values. The error bars show the SEM values.

#### Accession codes

GenBank accession codes of ACRs first reported in this article are listed in Table 3.

**Table 3.** Protein name abbreviations, GenBank accession numbers, source organisms, transcript names, and maxima of photocurrents action spectra of ACR homologs tested in this study

#	Protein name abbreviation	Accession	Organism	Transcript name	Spectral max. (nm)
1	*C1*ACR_561	MG831198	*Chroomonas* sp. (CCMP2293)	CAMNT 0022335561^*^	N/A
2	*Gc*ACR_197	MG831184	*Geminigera cryophila* (CCMP2564)	CAMNT 0021184197^*^	N/A
3	*Gc*ACR_201	MG831185	*Geminigera cryophila* (CCMP2564)	CAMNT 0021188201^*^	N/A
4	*Hp*ACR_213	MG831186	*Hanusia phi* (CCMP325)	CAMNT 0009606213^*^	N/A
5	*Ha*ACR_359	MG831187	*Hemiselmis andersenii* (CCMP1180)	CAMNT 0009587359^*^	N/A
6	*Hr*ACR_495	MG831188	*Hemiselmis rufescens* (PCC563)	CAMNT 0014430495^*^	N/A
7	*Psu*ACR_353	MG831189	*Proteomonas sulcata* (CCMP704)	CAMNT 0026606353^*^	520
8	*Ra*ACR_687	MG831190	*Rhodomonas abbreviata* (Caron Lab isolate)	CAMNT 0023412687^*^	520
9	*Rl*ACR_497	MG831191	*Rhodomonas lens* (RHODO)	CAMNT 0019250497^*^	520
10	RapACR/*Rs*ACR_665	MG831192	*Rhodomonas salina* (CCMP1319)	CAMNT 0012765665^*^	520
11	*R1*ACR_653	MG831193	*Rhodomonas* sp. (CCMP768)	CAMNT 0049478653^*^	470
12	*R1*ACR_6367	MG831194	*Rhodomonas* sp. (CCMP768)	CAMNT 0049496367^*^	480
13	*R2*ACR_041	MG831197	*Rhodomonas* sp. (CCAC1630)	IAYV-2051041^#^	N/A

* Transcripts from the MMETS project.

# Transcripts from the 1KP project.

#### Data availability

All data are available from the authors on reasonable request. Plasmids carrying functional ACR expression constructs are available through the nonprofit DNA distributor Addgene.

## Results

### Screening of ACR homologs

To explore the diversity of the ACR protein family and in search of better tools for optogenetics, we identified 13 new ACRs homologs in ongoing algal transcriptome sequencing projects (Keeling et al., 2014; Matasci et al., 2014), synthesized human codon-adapted polynucleotides encoding their transmembrane domains and expressed the resultant constructs in HEK293 cells as in-frame C-terminal EYFP fusions. The GenBank accession numbers, source organisms, transcript names, and protein names abbreviated by the first letters of the genus and species names and three or four last digits of the transcript codes are listed in Table 3.

**Figure 1.**
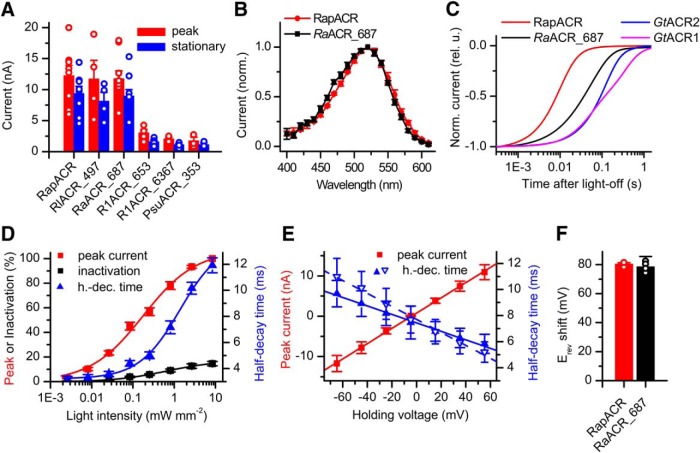
Screening of ACR homologs. ***A***, The amplitude of photocurrents generated by tested ACR homologs expressed in HEK293 cells in response to the first pulse of continuous light at the wavelength of the maximal sensitivity for each homolog (Table 3) at -60 mV at the amplifier output in standard solutions. The stationary current was measured at the end of a 1-s light pulse. The data are the mean values ± SEM (*n* = 3–10 cells). The data obtained in each individual cell are shown as empty circles. ***B***, The action spectra of photocurrents generated by RapACR (*Rs*ACR_665) and *Ra*ACR_687. The data are the mean values ± SEM (*n* = 4 and *n* = 8 scans, respectively). ***C***, The kinetics of the photocurrent decay after switching off the continuous light (1-s duration) at -60 mV. ***D***, The dependence of the normalized peak amplitude, inactivation, and half-decay time of RapACR photocurrents recorded in response to 1-s pulses of 520-nm light on the stimulus intensity. The data points are mean ± SEM (*n* = 5 cells). ***E***, The dependence of the peak amplitude (red) and half-decay time (blue) of RapACR photocurrents on the holding voltage corrected for the liquid junction potential. Filled symbols, solid lines, measurements using standard HEK293 solutions (Table 1); empty downward triangles, dashed line, measurements using neuronal solutions (Table 2). The data points are mean ± SEM (*n* = 6 cells). ***F***, The shifts of the reversal potential on partial replacement of Cl^–^ with Asp^-^ in the bath. The data are the mean values ± SEM (*n* = 3 and *n* = 5 cells for RapACR and *Ra*ACR_687, respectively).

Six of the tested homologs generated photocurrents in HEK293 cells. For three of the functional homologs, *Rs*ACR_665, *Rl*ACR_497, and *Ra*ACR_687, the mean peak current amplitude recorded at -60 mV with standard solutions (131 and 156 mM Cl^–^ in the pipette and bath, respectively; for other components, see Table 1) was >10 nA, whereas the other three homologs generated modest to small photocurrents (Fig. 1*A*). Although *Rs*ACR_665 and *Rl*ACR_497 were isolated from different algal species, their protein sequences differed only at three residue positions. Therefore, *Rl*ACR_497 was excluded from further analysis.

The spectral sensitivity of ACR homologs was determined by measuring the initial slope of current responses to 50-ms light pulses in the linear range of the intensity dependence, as described in Materials and Methods. Both *Rs*ACR_665 and *Ra*ACR_687 exhibited the spectral maxima at 520 nm (Fig. 1*B*); the spectral maxima for other homologs are listed in Table 3. The current decay kinetics after switching off the light was faster for both proteins than for *Gt*ACRs, and for *Rs*ACR_665, faster than for *Ra*ACR_687 (Fig. 1*C*). The current decay of *Rs*ACR_665 further accelerated on reducing the light intensity or shifting the voltage to more depolarized values (Fig. 1*D*,*E*). We nicknamed *Rs*ACR_665 “RapACR” to reflect its rapid channel kinetics.

To test the channels for permeability for Cl^–^, this ion in the bath was partially replaced with non-permeable aspartate, and a shift of the reversal potential (E_rev_) was determined by measuring the current-voltage dependencies, as described in Materials and Methods. On such replacement, the E_rev_ shifted to more positive values following the E_Cl_ (Fig. 1*F*), as has been shown for *Gt*ACRs (Govorunova et al., 2015). This observation confirmed that RapACR and *Ra*ACR_687 are anion-selective channels, as suggested by their sequence homology with earlier known cryptophyte ACRs.

### Comparison of RapACR with the second-generation Cl^–^-conducting CCR mutant iC++

**Figure 2.**
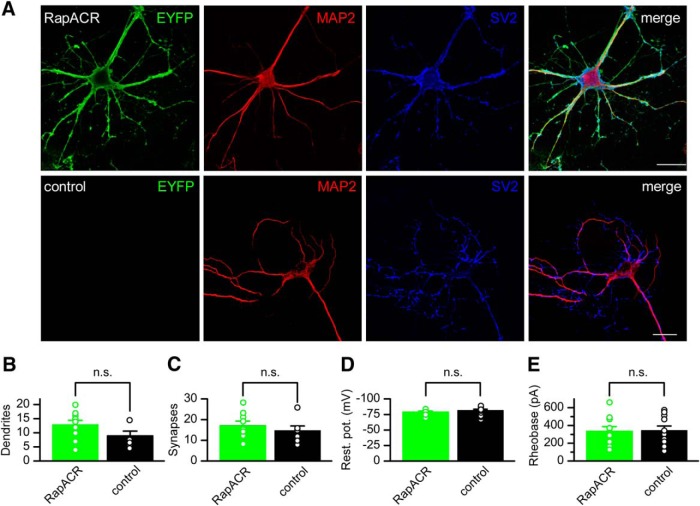
RapACR expression does not change morphologic and physiologic parameters of neurons. ***A***, Immunofluorescent images of neurons transduced with RapACR_EYFP fusion (top row) or control (non-transduced) neurons (bottom row) and stained with antibodies against EYFP (green channel), MAP2 (microtubule associated protein 2) as the dendrite marker (red channel), and SV2 (synaptic vesicle protein 2) as the synapse marker (blue channel). Scale bar, 20 µm. ***B***, ***C***, The number of dendrites per neuron and synapses per dendritic length of 20 µm, respectively. The data points are mean ± SEM (*n* = 10 and *n* = 7 cells for RapACR and control, respectively). ***D***, ***E***, The resting potential (abbreviated as rest. pot. on the *y*-axis in panel ***D***) and rheobase in the dark. The data points are mean ± SEM (*n* = 9 and *n* = 13 cells for RapACR and control, respectively, tested 8–14 d after transduction). Statistical significance was tested by the Mann–Whitney test.

To compare RapACR with the second-generation Cl^–^-conducting CCR mutant iC++ (Berndt et al., 2016) as optogenetic inhibitors, we expressed each construct under the ubiquitin promoter of the pFUGW vector (Lois et al., 2002) in cultured mouse hippocampal neurons using lentiviral delivery. RapACR showed predominantly plasma membrane localization (Fig. 2*A*). The ratio of the tag fluorescence in the plasma membrane to that in the cytoplasm was 13.9 ± 6.9 (mean ± SEM, *n* = 9 neurons). RapACR expression changed neither morphologic nor physiologic parameters of neurons in the dark, as compared to control (non-transfected) neurons (Fig. 2*B–E*).

**Figure 3.**
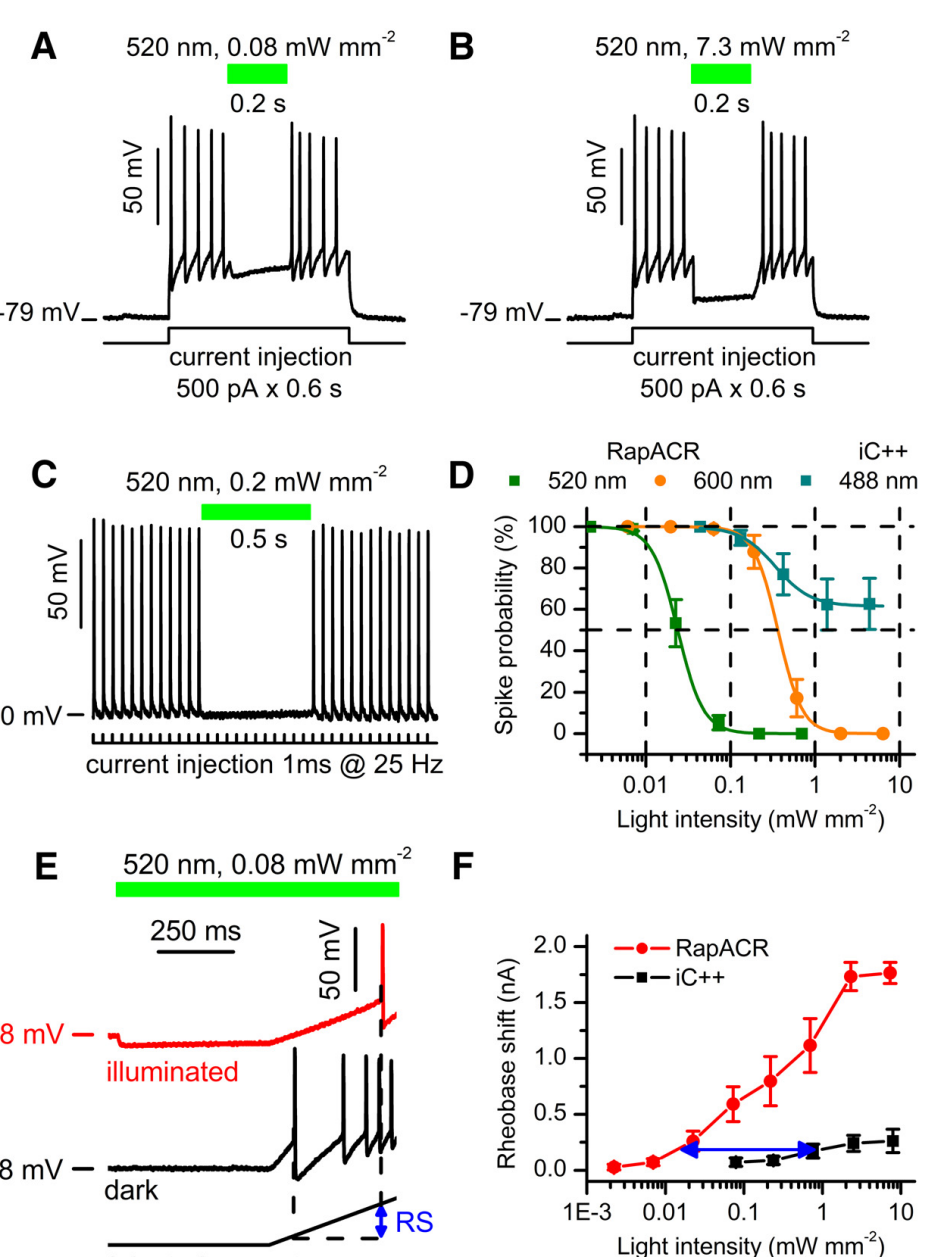
RapACR is more efficient for neuronal silencing than the second-generation engineered Cl^–^-conducting channelrhodopsin iC++. ***A***, ***B***, Photoinhibition of spiking in a neuron expressing RapACR at two different light intensities. Spiking was induced by depolarization of the membrane by prolonged current injection as shown at the bottom. The time course of illumination is shown as green bars on top. ***C***, A representative voltage trace recorded from a neuron expressing RapACR and stimulated with a train of 1-ms pulses of 2.5 nA delivered at 25 Hz. Passive response of the membrane (recorded under complete inhibition of spiking with light) was digitally subtracted. The time course of illumination is shown as a green bar. ***D***, The dependence of neuronal inhibition on the light intensity for RapACR and iC++ photoactivated at different wavelengths. The data points are mean ± SEM (*n* = 15 and *n* = 14 neurons for RapACR and iC++, respectively) approximated with a logistic function; fitting parameters are listed in Table 4. ***E***, Voltage traces recorded from a neuron transduced with RapACR and stimulated with a depolarizing current ramp (0–2 nA, 1 s; bottom trace) injected 500 ms after the onset of illumination (red) or in the dark (black). The blue arrow shows the photoinduced rheobase shift (RS). ***F***, The dependence of the rheobase shift on the light intensity in neurons transduced with RapACR or iC++. The current ramp was from 0 to 2 nA in 1 s. The blue arrow shows the difference in the light sensitivity between the two tested channels. The data points are mean ± SEM (*n* = 9 and *n* = 6 neurons for RapACR and iC++, respectively).

When tested in voltage clamp mode at the Cl^–^ gradient typical for the neuronal soma (4 and 135 mM Cl^–^ in the pipette and bath, respectively; for other solution components, see Table 2), RapACR generated hyperpolarizing photocurrents at the holding voltages above the E_Cl_ (∼-90 mV). Expression of RapACR in neurons enabled optical inhibition of their spiking (Fig. 3*A–C*). To determine the light sensitivity of optogenetic inhibition, virally transduced neurons were selected without regard to their tag fluorescence amplitude and stimulated by pulsed current. The probability of spiking (i.e., the percentage of current pulses that resulted in a spike) was measured in the dark and during illumination at various wavelengths and intensities. When activated at the wavelength of their maximal spectral sensitivity (520 and 470 nm, respectively), RapACR required similar energy densities to inhibit neurons as the earlier used *Gt*ACR2 (Fig. 3*D*, olive; Table 4). Furthermore, spiking in RapACR neurons could be completely inhibited even with 600-nm light (i.e., a wavelength 80 nm longer than the RapACR absorption maximum; Fig. 3*D*, orange). Among neurons transduced with iC++ and illuminated at the wavelength of its maximal sensitivity (488 nm), spiking was inhibited only in those with the brightest tag fluorescence, even at the highest light intensity tested. The mean data for iC++ neurons are shown in Figure 3*D*, dark cyan. We also measured the photoinduced rheobase shifts in neurons transduced with RapACR or iC++, as shown by the blue arrow in Figure 3*E* for RapACR. The half-maximal shift detected with iC++ was reached with RapACR at ∼50-fold lower light intensity (∼0.01 mW mm^−2^; Fig. 3*F*, blue arrow).

**Table 4.** Numerical parameters of logistic function fitting to the light sensitivity data

Figure	Dataset	x_0_	x_0_ error	*p*	*p* error	Chi^2^/DoF	*R* ^2^
3*B*	RapACR @ 520 nm	0.02376	0.00041	2.86833	0.22156	1.47822	0.99949
3*B*	RapACR @ 600 nm	0.36375	0.00229	3.05385	0.03368	0.11346	0.99996
3*B*	iC++ @ 488 nm	0.33928	0.02383	2.0744	0.27267	1.57038	0.99746
6*D*	*Gt*ACR1_C102A	0.00047	0.00002	1.05893	0.04073	2.29911	0.99822
6*D*	*Gt*ACR1	0.00299	0.00021	1.51384	0.14325	0.00101	0.99251
N/A	*Gt*ACR2 @ 470 nm	0.02948	0.00176	2.00325	0.23583	10.68705	0.99553
N/A	ZipACR @ 520 nm	3.2183	0.31733	1.47268	0.20552	19.85807	0.98616

### Using RapACR improves the time resolution of photoinhibition

*Gt*ACR2 has been shown to suppress spiking in neurons *in vitro* and *in vivo* at light intensities lower than those required by rhodopsin pumps and Cl^–^ conducting CCR mutants (Govorunova et al., 2015; Mohammad et al., 2017; Bergs et al., 2018). However, its photocurrent decays with a time constant of ∼40 ms (Govorunova et al., 2015), which is too slow for temporally precise inhibition (e.g., single spike suppression) of neurons firing at high frequencies. The performance of a faster variant, ZipACR, as a neuronal silencer has only been tested at the maximal light intensity (Govorunova et al., 2017b), and its light sensitivity remained unknown. We transduced neurons with the same amount of ZipACR virus as used for RapACR and measured the sensitivity to photoinhibition of randomly picked neurons without inspecting their tag fluorescence, as described in the previous section. We found that the sensitivity for ZipACR was ∼135-fold less than that for RapACR (Table 4). Thus, although ZipACR exhibits a faster channel decay than RapACR, its lower sensitivity decreases its utility as an optogenetic silencing tool as compared to RapACR.

**Figure 4.**
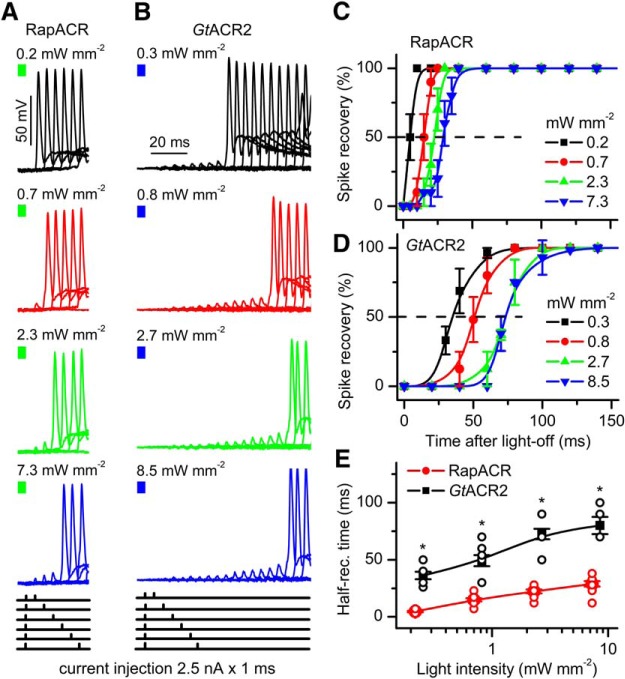
Using RapACR improves temporal resolution of neuronal silencing. ***A***, ***B***, Representative series of overlaid voltage traces recorded from neurons expressing RapACR or *Gt*ACR2, respectively, illuminated for 500 ms (the end of the light pulse is shown as a colored bar on top) at 520 or 470 nm, respectively, and stimulated with injection of a pair of 1-ms current pulses, the first of which was applied at the end of illumination, and the second of which, at an incrementally increased time after switching off the light (the injection protocols are schematically drawn at bottom; for *Gt*ACR2, only the first six protocols are shown). Passive response of the membrane (recorded under complete inhibition of spiking with light) was digitally subtracted. ***C***, ***D***, The time course of recovery of spiking after illumination measured as shown in panels ***A***, ***B*** for cells that could be inhibited with 1% light intensity. The data points are the mean values ± SEM (*n* = 10 and *n* = 8 neurons for RapACR and *Gt*ACR2, respectively). ***E***, The dependence of the time of 50% recovery of spiking on the light intensity calculated from the same cells as in ***C***, ***D***; *p < 0.001 (pairwise comparison of RapACR and *Gt*ACR2 data at each intensity by the Mann–Whitney test). Data obtained in each individual neuron are shown as empty circles.

The rate of membrane repolarization after switching off the light depends not only on the rate of channel closing, but also on the photocurrent amplitude, both of which are light dependent. We measured the light intensity dependence of the time required for spiking recovery after photoinhibition for RapACR and *Gt*ACR2. Most rodent hippocampal neurons cannot be repetitively stimulated at frequencies higher than 50 Hz, although they are capable of generating short high-frequency bursts of spikes (Graves et al., 2012). To determine accurately the time required for the recovery of spiking after photoinhibition mediated by ACRs, we injected a pair of depolarizing current pulses per trace. The first pulse was applied precisely at the end of illumination to test whether spiking was inhibited at each particular light intensity. The second was injected at an incrementally varied time after switching off the light to test for spiking recovery. Figure 4*A*,*B* shows representative series of overlaid voltage traces recorded from neurons expressing RapACR or *Gt*ACR2, respectively. In both cases, the rate of recovery depended on the light intensity. Although the mean light sensitivities of neurons transduced with RapACR or *Gt*ACR2 were similar (Table 4), there was variability between individual cells. Therefore, for more accurate comparison of the recovery rates in neurons transduced with RapACR or *Gt*ACR2, we selected only those neurons that required approximately the same threshold light intensity (1% of the maximum) of inhibition. Figure 4*C*,*D* shows the time course of spiking recovery for such neurons, and Figure 4*E*, the dependence of the half-recovery time on the light intensity. RapACR enabled faster recovery than *Gt*ACR2 at all tested light intensities. At the lowest light intensity sufficient for silencing, RapACR enabled complete restoration of spiking already at 10 ms after switching off the light, whereas with *Gt*ACR2, 80 ms was required.

### Mutagenetic alteration of ACR photocurrent kinetics

**Figure 5.**
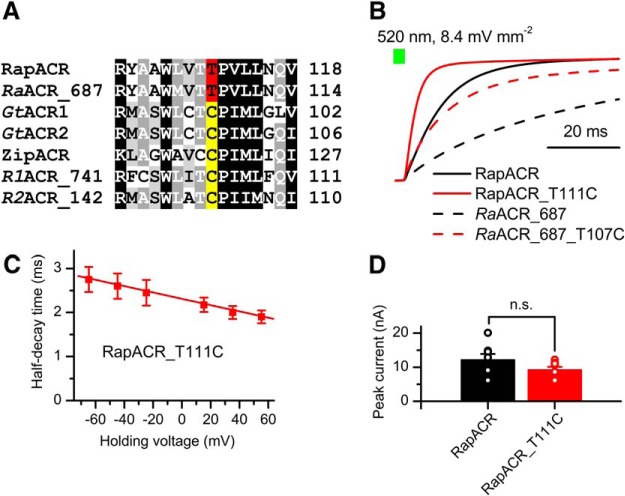
Characterization of the fast RapACR_T111C and *Ra*ACR_687_T107C mutants. ***A***, A ClustalW alignment of the 3^d^ transmembrane helix around the Cys-128 position (*Cr*ChR2 numbering) from indicated ACRs. The Thr residues found in this position are highlighted red, Cys residues, yellow. ***B***, Normalized photocurrent decay after 1-s illumination recorded from wild-type RapACR (black, solid) and *Ra*ACR_687 (black, dashed), and the corresponding T111C (red, solid) and T107C (red, dashed) mutants. ***C***, The dependence of the half-decay time of RapACR_T111C photocurrent on the holding voltage corrected for the liquid junction potential. The data points are the mean ± SEM (*n* = 4 cells). ***D***, Peak photocurrent amplitude at -60 mV. The wild-type data are from Figure 1*A*, the data for RapACR_T111C are the mean values ± SEM (*n* = 8 cells). Statistical significance was tested by the Mann–Whitney test. The values obtained in individual cells are shown as open circles.

In RapACR and *Ra*ACR_687, the position of Cys-102 (*Gt*ACR1 numbering) is occupied with Thr (Fig. 5*A*). Replacement of this residue (Thr111) with Cys in RapACR led to acceleration of the current decay (Fig. 5*B*, red solid line), so that at the positive voltages its half-time approached 2 ms (Fig. 5*C*) matching that of ZipACR, the fastest so far found wild-type ACR (Govorunova et al., 2017b). The peak current amplitude of the T111C mutant was not significantly different from that of the wild-type RapACR (Fig. 5*D*). The corresponding T107C mutation in *Ra*ACR_687 also accelerated the current decay (Fig. 5*B*, red dashed line) from t_1/2_ 35 ± 4 ms in the wild type (*n* = 10 cells) to 12 ± 3 ms in the mutant (*n* = 5 cells).

**Figure 6.**
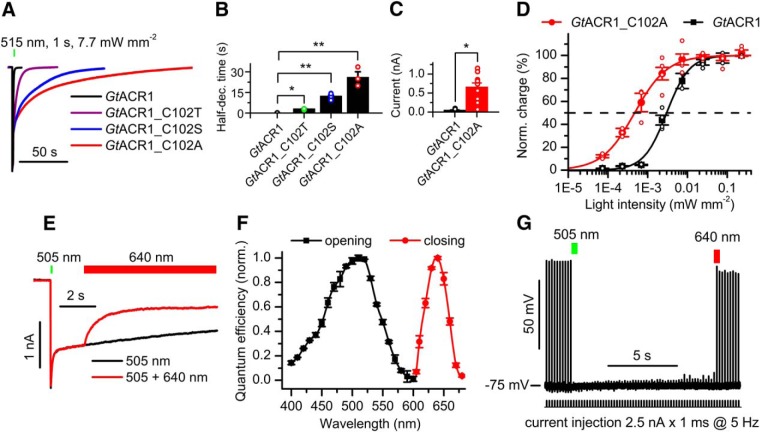
Characterization of the *Gt*ACR1_C102X mutants and the use of *Gt*ACR1_C102A as a bistable photochromic silencing tool. ***A***, Normalized photocurrent traces recorded at -60 mV in HEK293 cells. ***B***, The half-time of the slow decay phase. The data are mean ± SEM (*n* = 4–12 cells; for exact numbers, see Table 5); *p < 0.05, **p = 0.01; Kruskal–Wallis test with Bonferroni correction. ***C***, The amplitudes of stationary photocurrents in HEK293 cells at -60 mV after 200-s illumination. The wavelengths were 515 and 505 nm for *Gt*ACR1 and *Gt*ACR1_C102A, respectively. The data are mean ± SEM (*n* = 13 and *n* = 12 cells, respectively); *p < 0.001; Mann–Whitney test; see Table 6 for full statistics. ***D***, The dependence of electrical charge transferred across the membrane during 100-s illumination at -60 mV. The data are mean ± SEM (*n* = 5 and *n* = 6 cells for *Gt*ACR1 and *Gt*ACR1_C102A, respectively). The fitting parameters are listed in Table 4. ***E***, Opening and partial closing of *Gt*ACR1_C102A with light at -60 mV. ***F***, The action spectra of *Gt*ACR1_C102A opening and closing. The data points are mean ± SEM (*n* = 8 scans). ***G***, Bidirectional optical control of neuronal spiking with *Gt*ACR1_C102A. Passive response of the membrane (recorded under complete inhibition of spiking with light) was digitally subtracted. In panels ***B–D***, also the data obtained in each individual cell are shown as empty circles.

Instead of precise light control in the ms time domain, some optogenetic experiments require prolonged inhibition after a short light pulse. It has been shown that substitution of Cys102 in *Gt*ACR1 with Ala leads to a dramatic extension of the second phase of the photocurrent decay (Sineshchekov et al., 2015). On stimulation with continuous light pulses, the amplitude of the slow decay phase in the *Gt*ACR1_C102A mutant was ∼50% at the maximal intensity (Fig. 6*A*, red), whereas at low light intensities this phase dominated the decay. Mutagenetic replacement of Cys102 with Thr or Ser caused a lesser effect than replacement with Ala (Fig. 6*A*,*B*; see Table 7 for full statistics). At 0.2 µW mm^−2^, the C102A mutant generated ∼13 times larger stationary currents than the wild type (Fig. 6*C*). For 100-s illumination, the C102A mutant required approximately seven times less light than the wild type to reach the half-saturating level of charge transfer (Fig. 6*D*). Illumination with red light caused partial closing of the *Gt*ACR1_C102 channel (Fig. 6*E*). The action spectrum of channel opening (Fig. 6*F*, black) corresponded to the absorption spectrum of *Gt*ACR1_C102A expressed and purified from *Pichia* (Sineshchekov et al., 2016) and was slightly blue-shifted from that of the wild-type (the maximum at 505 vs 515 nm). The action spectrum of channel closing peaked at 640 nm (Fig. 6*F*, red).

**Table 5.** Numbers of independent tests (*n* values)

Figure	Variant	*n*
1*A*	RsACR_665	8
	RlACR_497	4
	RaACR_687	10
	R1ACR_653	4
	R1ACR_6367	5
	PsuACR_353	3
6*B*	*Gt*ACR1	12
	*Gt*ACR1_C102T	13
	*Gt*ACR1_C102S	7
	*Gt*ACR1_C102A	3
7*E*	*Ra*ACR_687	10
	*R2*ACR_142	12
	RapACR	5
	ZipACR	8
	*Ra*ACR_687_T107A	5
	*R2*ACR_142_C102A	6
	RapACR_T111A	5
	ZipACR_C119A	7
7*F*	*Ra*ACR_687_T107A	5
	*R2*ACR_142_C102A	6
	RapACR_T111A	7
	ZipACR_C119A	7

**Table 6.** Results of independent-samples Mann–Whitney tests

Figure	Variant	Total	Mann–Whitney *U*	Wilcoxon *W*	Test statistic	SE	Standard test statistic	Asymptotic Significance (two-sided test)	Exact Significance (two-sided test; *p* value)
4*E*	0.2 (0.3)	18	80.000	116.000	80.000	10.865	3.682	0.000	0.000
	0.7 (0.8)	18	80.000	116.000	80.000	11.179	3.578	0.000	0.000
	2.3 (2.7)	18	80.000	116.000	80.000	11.050	3.620	0.000	0.000
	7.3 (8.5)	18	80.000	116.000	80.000	10.978	3.644	0.000	0.000
5*D*		16	17.000	53.000	17.000	9.522	-1.575	0.115	0.130
6*C*		25	152.000	230.000	152.000	18.385	4.025	0.000	0.000
2	*B*	17	20.000	48.000	20.000	10.190	-1.472	0.141	0.161
	*C*	17	29.000	57.000	29.000	10.197	-0.588	0.556	0.601
	*D*	22	43.500	134.500	43.500	14.941	-1.004	0.315	0.324
	*E*	22	74.500	165.500	74.500	14.971	1.069	0.285	0.292
8	*B*	41	186.500	339.500	186.500	37.247	0.470	0.638	0.668
	*C*	41	212.000	365.000	212.000	37.649	0.212	0.832	0.870
	*D*	23	57.000	148.000	57.000	16.069	-0.498	0.619	0.648
	*E*	23	67.000	158.000	67.000	16.125	0.124	0.901	0.927

**Table 7.** Results of independent-samples Kruskal–Wallis test with Bonferroni correction

Figure	Variant	Test statistics	SE	Standardized test statistics	Significance
6*B*	C102T vs Wild type	-12.500	4.102	-3.047	0.002
	C102S vs Wild type	-13.500	3.637	-3.712	0.000
	C102A vs Wild type	-18.500	4.936	-3.748	0.000

**Figure 7.**
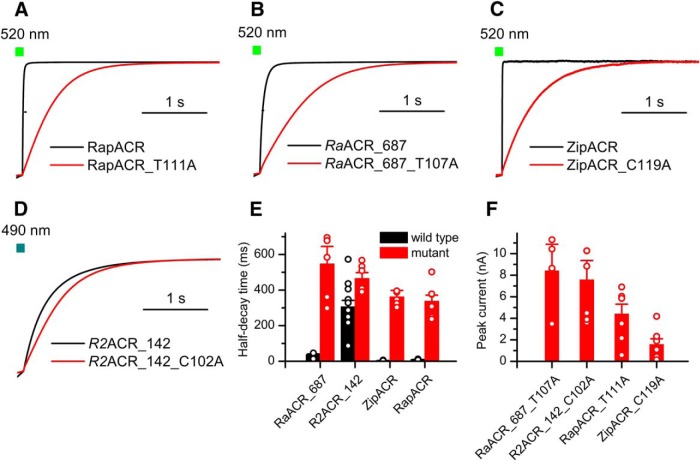
Biophysical characteristics of slow ACR mutants. ***A–D***, Normalized photocurrent decay after 1-s illumination recorded from wild-type ACRs (black) and their respective mutants in which the residue homologous to Cys-102 (*Gt*AR1 numbering) was mutated to Ala (red). ***E***, ***F***, Photocurrent half-decay times (***E***) and peak photocurrent amplitudes (***F***) measured in wild-type ACRs and their indicated mutants. The data points are mean ± SEM (*n* = 5–12 cells; for exact numbers, see in Table 5).

**Figure 8.**
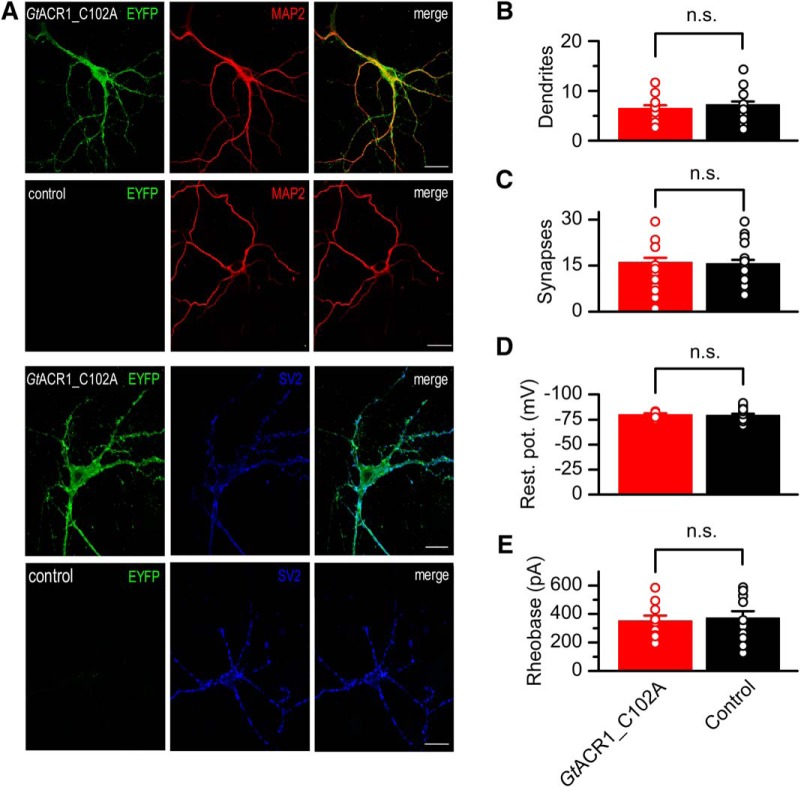
GtACR1_C102A expression does not change morphologic and physiologic parameters of neurons. ***A***, Immunofluorescent images of neurons transduced with *Gt*ACR1_EYFP fusion (odd rows) or control (non-transduced) neurons (even rows) and stained with antibodies against EYFP (green channel), MAP2 (microtubule associated protein 2) as the dendrite marker (red channel), and SV2 (synaptic vesicle protein 2) as the synapse marker (blue channel). Scale bar, 20 µm. ***B***, ***C***, The number of dendrites per neuron and synapses per dendritic length of 20 µm, respectively. The data points are mean ± SEM (*n* = 17 and *n* = 24 cells for *Gt*ACR1_C102A and control, respectively). ***D***, ***E***, The resting potential (abbreviated as rest. pot. on the *y*-axis in panel ***D***) and rheobase in the dark. The data points are mean ± SEM (*n* = 10 and *n* = 13 cells for *Gt*ACR1_C102A and control, respectively, tested 8–14 d after transduction). Statistical significance was tested by the Mann–Whitney test (see full results in Table 6).

Replacement of Thr111 with Ala in RapACR slowed the current decay, as did the corresponding C102 mutation in *Gt*ACR1, but the effect was much less pronounced (Fig. 7*A*). We also tested the corresponding Ala replacement mutants in *Ra*ACR_687 and two other ACRs that generate large photocurrents, ZipACR and *R2*ACR_142 (Govorunova et al., 2017b). The results (Fig. 7*B–F*) show that although the mutation led to an extension of the decay time in all tested mutants, none of them exhibited slower decay than the *Gt*ACR1_C102A mutant. Therefore, we used the latter to inhibit neuronal spiking. We cloned *Gt*ACR1_C102A in the same vector backbone as used for wild-type ACRs and expressed it in cultured mouse hippocampal neurons by lentiviral delivery. No changes in morphologic or physiologic parameters of neurons in the dark were detected on *Gt*ACR1_C102A expression (Fig. 8). A brief light pulse of green (505 nm) light was sufficient to inhibit neuronal spiking for at least 10 s after switching the light off (Fig. 6*G*). In neurons transduced with *Gt*ACR1_C102A, spiking was rapidly restored with a pulse of red (640 nm) light (Fig. 6*G*).

## Discussion

Both natural and engineered Cl^–^-conducting channelrhodopsins can be considered as silencing tools more physiologic than rhodopsin pumps, since they “shunt” the membrane potential to the E_Cl_ (which in most neurons is close to the resting potential), and therefore prevent non-physiologic changes in the intracellular Cl^–^ concentration, which have been reported when using the Cl^–^ pump halorhodopsin (Raimondo et al., 2012; Alfonsa et al., 2015).

We show that the light sensitivity of some cryptophyte ACRs as neuronal silencers exceeds not only that of rhodopsin pumps (Govorunova et al., 2015; Mohammad et al., 2017; Bergs et al., 2018) but also that of improved, second-generation engineered Cl^–^-conducting channelrhodopsins, which makes cryptophyte ACRs the most efficient silencing tools available. When tested side-by-side, RapACR enabled complete silencing of neurons at a wavelength 110 nm more red-shifted than that of the peak absorbance of iC++, at which the latter tool failed to suppress spiking in >50% of randomly picked neurons (Fig. 3*D*). Engineered Cl^–^-conducting channelrhodopsins have recently been referred to as “engineered ACRs” (eACRs) in the literature (Wietek et al., 2017), but this term is misleading, because chlorophyte CCRs from which they were derived differ from natural cryptophyte ACRs not only in the mutated positions, but form a structurally distinct protein family (Govorunova et al., 2017b).

So far, studies using *Gt*ACRs as optogenetic inhibitors of behavior have been conducted in worms (Bergs et al., 2018; Xu et al., 2018), flies (Mauss et al., 2017; Mohammad et al., 2017; Steck et al., 2018), zebrafish (Mohamed et al., 2017), and mouse (Mahn et al., 2017; Forli et al., 2018; Li et al., 2018; Mardinly et al., 2018; Wei et al., 2018). ACRs are also expected to enable silencing of all those types of mammalian neurons in which inhibition has been demonstrated with engineered light-gated Cl^–^-conducting channels (Wietek et al., 2015; Berndt et al., 2016; Iyer et al., 2016; Kim et al., 2016; Takahashi et al., 2016; Chung et al., 2017) and possibly many more. Switching to highly conductive natural ACRs as optogenetic silencing tools will help to: (1) minimize light intensities and thus prevent overheating of the tissue; and (2) decrease expression levels to avoid possible detrimental effects on the physiology of recipient cells, as compared to other available tools.

The kinetics of channel closing of *Gt*ACRs enables single-spike suppression in neurons firing at moderate rates (Govorunova et al., 2015) but limits temporal precision of spike suppression in neurons spiking at high frequencies. Faster natural ACRs that generate comparably large photocurrents, such as ZipACR (Govorunova et al., 2017b) or RapACR reported here, are capable of single-spike suppression even in high-frequency firing neurons. The theoretically estimated upper limit of firing frequency at which precise neuronal silencing with ZipACR is possible is 200 Hz, which, however, has not yet been confirmed experimentally (Govorunova et al., 2017b). Although ZipACR and RapACR generated equally large photocurrents in HEK293 cells (Govorunova et al., 2017b; and this study, respectively), ZipACR required much more light to inhibit cultured mammalian neurons than RapACR or *Gt*ACR2 (Table 4). The latter observation is consistent with the data obtained in *C. elegans* muscles, where the performance of ZipACR was also inferior to that of *Gt*ACR2 (Bergs et al., 2018). This discrepancy between the results obtained with the same tool in different cell types emphasizes the importance of cell-specific tool testing.

The limit for silencing with RapACR that we determined by measuring the recovery rate at the minimal light intensity sufficient to fully suppress spiking is at least 100 Hz (Fig. 4), and this number may be an underestimate, because we measured the recovery only in 5-ms time increments. Further acceleration of ACR channel kinetics, and thereby, a further increase in temporal precision of inhibition, can be achieved by site-directed mutagenesis, as we show here for the RapACR_T111C mutant (Fig. 5). Importantly, fast cryptophyte ACRs permit temporally precise neuronal silencing at low light intensities, in contrast to rhodopsin pumps that also generate fast photocurrents but require strong light. However, when temporally precise photoinhibition using ACRs is needed, it is important to take into consideration that the time required for spiking recovery after photoinhibition depends on the light intensity and is minimal at the minimal intensity sufficient to suppress spiking (Fig. 4*E*). Improved temporal resolution can also be achieved by reducing ACR expression level (although this will also lead to reduction of the light sensitivity).

ACRs with dramatically extended channel opening time could be engineered to boost their light sensitivity and convert them to step-function tools (Fig. 6). Despite only partially overlapping residue conservation patterns between ACRs and CCRs (Govorunova et al., 2017a), the C102 position in *Gt*ACR1 (corresponding to Cys128 in *Cr*ChR2) appears to be a common regulator of channel kinetics in both channelrhodopsin families. *Gt*ACR1_C102A functions as a bistable photochromic step-function tool for spike inhibition as does *Cr*ChR2_C128A for spike generation.

Natural ACRs and their mutants reported here enable efficient neuronal silencing on time scales both shorter and longer than that currently available, which widens the range of potential applications of these powerful optogenetic silencing tools.

## Synthesis

Reviewing Editor: Kirill Martemyanov, The Scripps Research Institute Decisions are customarily a result of the Reviewing Editor and the peer reviewers coming together and discussing their recommendations until a consensus is reached. When revisions are invited, a fact-based synthesis statement explaining their decision and outlining what is needed to prepare a revision will be listed below. The following reviewer(s) agreed to reveal their identity: Adam Cohen.

Synthesis.

Both reviewers felt that the report contains interesting information introducing new tool for inhibitory optogenetics with advantageous kinetics and light sensitivity. Several variants are made and optimized for performance. It is likely that these reagents will serve as useful tools for optogenetic experiments.

During discussion, the reviewers agreed that most of their suggestions were technical in nature and should be straightforward to address by editing and clarifications in the text. However, one major limitation that was brought up and agreed upon by the reviewers was that for the anion chanelorhodopsins to be useful tools for optogenetics, their performance in the in vivo applications and/or native networks and circuits would need to be documented. There was a consensus that requesting to include such data in the revised manuscript may fall outside of the scope of the report. Nevertheless, some demonstration of probe performance in at least a model neuronal network, e.g. by evoking a hyperexcitable state in culture (e.g. by adding Gabazine; or by going to low Mg^2+ and high K+) and showing an inhibitory effect on spontaneous network activity would be imperative. Additional specific issues that need to be resolved are listed below in specific reviewers' comments.

Reviewer #1

This manuscript reports the engineering and characterization of cryptophyte anion channelrhodopsins with high photosensitivity and different kinetic properties. The authors first identified RapACR, an anion channlerhodopsin with high photosensitivity and fast closing kinetics from the existing genomic sequencing data. They further showed that a single mutation T111C could further accelerate its closing kinetics without significantly compromising the photosensitivity. Their comparison of RapACR and zipACR, a previously reported fast ACR, found that while zipACR showed moderately faster closing kinetics, RapACR has two-order-of-magnitude higher photosensitivity. They also engineered a previously reported ACR, GtACR1, to be a bistable step-function tool. The mutant GtACR1 could be turned on with 505-nm light and off with 640-nm light. The majority of the experiments were done in cultured neurons with good statistics. Overall, this work is a valuable contribution to the optogenetics field. The authors should be commended for a thorough and clear experimental section and careful data analysis.

Minor questions:

1. The authors mentioned that “RapACR showed predominantly plasma membrane localization”. In my previous experience with GtACRs, intracellular aggregates could be clearly observed. zipACR, however, showed much better membrane localization than GtACR1 and 2. In the confocal images, it appears that RapACR (Fig.2A) trafficks better than GtACR1 (Fig.6A). Can the authors comment on the trafficking of RapACR with greater details? Does the addition of retinal in the culture media facilitate trafficking?

2. A theoretical merit of RapACR is that it allows the elimination of a single spike at faster frequency. However, the experimental demonstration of such an important advantage is not presented in this manuscript. What is shown in Fig.3A is the elimination of multiple spikes at 25Hz. In fact, in Govorunova2015, it was already shown that GtACR could inhibit a single spike at 25Hz (Govorunova2015, Fig.4C). Is there any experimental limit that prevented the authors from showing such data?

In the abstract, the authors write that “The upper limit of the time resolution of neuronal silencing with RapACR determined by measuring the dependence of spiking recovery after photoinhibition on the light intensity was 100 Hz, whereas that with the faster of the two G. theta anion channelrhodopsins was 22 Hz.” Is this statement at odds with the Govorunova2015? Besides, the readers may get get the impression that this manuscript has directly demonstrated single-spike elimination at 100 Hz. I recommend the author to rephrase this sentence as “the theoretical upper limit of the time resolution...”

3. The last sentence in the abstract concludes: “these molecules extend the time domain of optogenetic neuronal silencing without the need to increase the light intensity”. “without the need to increase the light intensity” reads confusing as the context is not clear. The authors could simply say “these molecules extend the time domain of optogenetic neuronal silencing while retain the high light sensitivity of GtACRs”

Fig. 1e: Is this experiment done when the chloride reversal potential is 0 mV? That's what it looks like from the x-axis, but not clear from the figure caption. It would be of some interest to know how the half-decay time varies with membrane voltage when physiological Cl- concentrations are used: is the half-decay time set by the absolute membrane voltage, or the difference between V_m and the chloride reversal potential?

Fig. 4B: The legends say ‘mV mm^{-2}’. mV --&gt; mW.

Line 398: swithing --&gt; switching

Line 468: silences --&gt; silencers

Reviewer #2

In this research article the Authors describe novel variants of anion channelrhodopsins with rapid kinetics and increased sensitivity to light. Optogenetic inhibition provides a powerful approach to study the contribution of distinct neurons and specific circuits both in basic and applied Neuroscience and the identification of novel optical silencers represent thus an important and valuable addition to the field.

The authors screen and validate several variants of anion channelrhodopsins in vitro using both HEK293 cells lines and hippocampal neuronal cultures and the results are clearly reported.

I have a few observations that would be worth clarifying/discussing before this manuscript reaches its final form:

1) In line 166 the authors state that patch clamp experiments were carried out 5-14 days post transduction. However, legends for Figure 2 and 8 show that data were collected between 8-14 days. This is confusing and need to be clarified in light of the differences for rheobase magnitude vs days of cultivation.

2) There is no indication about the lentiviral infection protocol (amount of virus, method of delivery etc...). It is also important to report the titer of the virus used.

3) What is the percentage of fluorescent neurons being efficiently silenced during light? Did the light treatment have any effect on the firing of non infected neurons?

4) Did any of the variants tested show any cell specificity? The authors refer to Graves et al., 2012 which show differences in the morphological and electrophysiological properties of pyramidal cells in CA1 and subiculum. The authors should discuss how neurons in their cultures were classified and clustered.

5) The protocols used for stimulation are not always clear and need to be specified in the methods section as well as for each figure.

6) The manuscript will largely benefit from an additional series of experiments showing the efficiency of these anion channelrhodpsin variants in silencing neuronal firing over a range of stimulation frequencies in vivo or in a model of elevated neuronal network excitability.

Minor points:

ABSTRACT:

• Line 9: The authors should use RsACR_665 or explain the nickname RapACR as appears in line 345.

• Line 16: Change G.theta with Guillardia theta.

INTRODUCTION:

• The references listed in line 43 do not directly relate high expression levels and high light intensities to the efficiency of neuronal silencing but rather report the need to suppress larger brain areas to assess their contribution to specific behaviors. The authors should consider rephrasing or cite more direct evidences for the efficacy of proton/chloride pumps in silencing local tissue.

MATERIALS AND METHODS:

• In Line 198, spell PBS as phosphate-buffered saline.

• For each antibody provide the catalogue number.

• In line 230 define s.e.m and adopt only one acronym throughout the manuscript.

• Line 249, correct ClampEx with Clampex.

RESULTS:

• The values reported in Line 331 do not match with those reported in Table 1.

• Lines 365-368: The authors only used one frequency of stimulation, it is not clear how this can relate to an in vivo scenario. Consider rephrasing.

• Line 379: Figure 3C does not show the rheobase shift in neurons transduced with RapACR or iC++.

• Line 381: Panel 3F does not exist.

• Line 391-392: To compare the sensitivity to photoinhibition of neurons transduced with different viruses, the viral titer rather than the amount is a key. Both information however are not specified.

• Line 407: Figure 4B & C do not show overlaid voltage traces.

• Line 426-428: Where are these data shown?

• Lines 456-459: The authors should move these two sentences to the previous paragraph.

FIGURES:

• Line 381 refers to Figure 3F however this panel is missing.

• Figure 2 and Figure 8: Define ‘Rest. pot.’ in the figure legend, or do not abbreviate.

• Figure Legend 3 does not have a title.

• In Figure 3A and C indicate the resting membrane potential, add scale for current injection.

• In Figure 4A and B specify the current injection protocol. Color traces can be changed to match the curves in C and D.

• Figure 6G: is this neuron spontaneously firing? Otherwise indicate the protocol used for firing stimulation.

• Data obtained for each individual neurons should be shown in the bar graphs of Figure 2 and 8.

• Line 795: NMDG does not appear in Table 2.
